# Power, interests, and maternal health care: a political economy analysis of service delivery redesign in Kenya

**DOI:** 10.1093/heapol/czaf111

**Published:** 2025-12-18

**Authors:** Jacinta Nzinga, Easter Olwanda, Kennedy Opondo, Hillary Kimutai, Jan Cooper, Brian Arwah, Benjamin Tsofa, Edwine Barasa, Kevin Croke

**Affiliations:** Department of International Public Health, Institute for Resilient Health Systems, Liverpool School of Tropical Medicine, Liverpool L3 5QA, United Kingdom; Health Economics Research Unit, KEMRI Wellcome Trust Research Programme, P.O Box 43460-00100, 197 Lenana Place, Hurlingham, Nairobi, Kenya; Institute for Human Development, The Aga Khan University, P.O Box 30270-00100, Nairobi, Kenya; Department of Global Health and Population, Harvard TH Chan School of Public Health, Bostin, MA 02115, United States; Centre for Excellence in Women and Child Health, The Aga Khan University, P.O Box 30270-00100, Nairobi, Kenya; Department of Global Health and Population, Harvard TH Chan School of Public Health, Bostin, MA 02115, United States; Health Economics Research Unit, KEMRI Wellcome Trust Research Programme, P.O Box 43460-00100, 197 Lenana Place, Hurlingham, Nairobi, Kenya; Health Economics Research Unit, KEMRI Wellcome Trust Research Programme, P.O Box 43460-00100, 197 Lenana Place, Hurlingham, Nairobi, Kenya; Health Economics Research Unit, KEMRI Wellcome Trust Research Programme, P.O Box 43460-00100, 197 Lenana Place, Hurlingham, Nairobi, Kenya; Nuffield Department of Medicine and Department of Paediatrics, University of Oxford, Oxford OX3 7BN, United Kingdom; Department of Global Health and Population, Harvard TH Chan School of Public Health, Bostin, MA 02115, United States

**Keywords:** health systems reforms, service delivery redesign, political economy analysis, maternal and newborn

## Abstract

The maternal and newborn health (MNH) service delivery redesign (SDR) in Kakamega County, Kenya, represents the country's first system-level reorganization of MNH services. The reform aimed to improve care quality and reduce mortality by centralizing delivery services at designated hubs. Using a political economy lens, we examined how ideology, political dynamics, and institutional structures shaped the agenda-setting, adoption, implementation, and sustainability of SDR. We drew on data from document reviews, stakeholder analysis, semi-structured interviews, and non-participant observation to assess the structural, contextual, and institutional factors influencing the reform. Ambiguity around SDR's purpose contributed to the community's uncertain engagement characterized by neither full endorsement nor resistance, highlighting the need for clearer communication and participation to build ownership. The interaction between formal institutions (county health governance and partnership frameworks) and informal norms (trust, shared interpretation, and relational coordination) created early momentum for implementation, particularly among health system actors. However, limited financial capacity and unclear alignment with national policy priorities undermined progress and long-term viability. Kakamega's experience demonstrates how political incentives, devolved autonomy, and local institutional context jointly shape reform outcomes. Achieving successful implementation of system-level reforms requires integrating local political leadership, strengthening community engagement, aligning with evolving national policies, and securing predictable financing. This study provides practical lessons for future MNH and system-level reforms in Kenya and similar decentralized, resource-constrained settings. Lessons include the importance of balancing formal and informal institutions to ensure both political feasibility and enduring impact.

Key messages

- We examined how political and institutional dynamics of health system reforms shape the implementation processes of maternal and newborn health (MNH) reforms in a devolved, resource-limited setting. We investigated how Service Delivery Redesign (SDR) implementation in Kakamega can enhance care quality and reduce mortality, providing lessons in other similar settings.
- Kakamega's SDR implementation was supported by a combination of structured county health partnership agreements and informal relationship-building among key decision-makers and stakeholders. However, unclear communication about the reform's goals hindered community support, underscoring the need for robust engagement strategies.
- Limited funding and shifting national health policies posed significant challenged to SDR's implementation progress and long-term sustainability, highlighting the vulnerability of decentralized reforms in resource-constrained environments.
- This work provides lessons for implementing MNH reforms. Success requires political commitment, community ownership supported by clear communication, sustainable financing, and alignment with national policies.

## Introduction

Innovative ways of delivering health services can improve equity, quality, and health outcomes if widely and appropriately implemented (Roder-DeWan et al. 2023). Service delivery redesign (SDR) is one such innovation; it is a new model of care which involves reorganizing and strengthening existing services and care pathways to maximize quality care and optimize health outcomes (Nimako et al. 2021, Roder-DeWan et al. 2023). However, the implementation of SDR is not solely a technical process; it is also shaped by socio-economic and political factors that influence policy design, adoption, and reform execution. For instance, SDR implementation requires both technical and political leadership to drive high-quality and respectful care, as well as evidence-based contextually appropriate strategies to address inequity and improve the quality of services provided (Kunzler 2016)

SDR approaches are often framed as ‘system reforms’ reflecting its purpose as a deliberate improvement of the health system. SDR occures through a continuous reform process acting upon complex interdependent systems, rather than a discrete policy change implemented at a single point in time (Roder-DeWan et al. 2023). The process of implementing SDR can differ depending on context, and on the health system's capacity to innovate and ensure the provision of the right care available at the right level and at the right time. In Kakamega County, Kenya, the government implemented a maternal and newborn health (MNH) service delivery redesign aimed at improving maternal and newborn survival by shifting deliveries, from lower-level facilities which have limited capacity to address delivery complications to adequately prepared, designated delivery hospitals. Given the complexity of the process, SDR requires the engagement of multiple actors across the system and coordination, continuity, and consistent implementation across multiple domains of the health system (i.e. clinical care, financing, transport, and human resources). Furthermore, health system change processes are inherently political, as they entail contestation over power and resources. Political economy analysis (PEA) can be used to understand the politics underlying seemingly technical health reforms, identify who the most important stakeholders are, how they influence the implementation process, and ultimately how political economy factors affect reform outcomes (Andreas et al. 2022).

A political economy approach prompts us to ask who the key actors are in a given policy domain; it requires us to consider ‘who wins and who loses out’ from policy change and pushes us to analyse how those who stand to lose may resist change. In recent years, there has been increasing recognition of the role of politics in health system reforms, leading to an expansion of tools and approaches for undertaking PEA (Sparkes et al. 2019). These tools emphasize the central role of actors and stakeholders alongside proposed methods for capturing their roles and positions vis-à-vis proposed reforms (such as stakeholder mapping or social network analysis, Reich 2019). Related approaches focus on the politics of how problems are identified, debated, and refined by policy actors (‘agenda setting’) as well as the politics of policy adoption and implementation. Analysts have emphasized the importance of ensuring that the changes sought are grounded in contextual realities, based on what is both politically feasible and technically sound (Moncrieffe and Luttrell 2005).

Our analysis of the political economy of Kakamega SDR reform examines how the complex relationship between actors, interest groups, and formal and informal institutions influenced SDR's agenda-setting, adoption, implementation, and sustainability. Specifically, we explore the extent to which structural, contextual, and institutional actors’ motivations influenced the SDR implementation process. Kakamega is the first region in Kenya to implement a deliberate system-level reorganization of MNH services, and therefore, the lessons learned from this analysis may provide valuable insights for similar system-level reforms in other settings.

### The Kakamega service delivery redesign intervention and its rationale

More than half of preventable maternal and neonatal deaths in Low Middle Income Countries are estimated to be due to poor quality care during childbirth rather than inadequate health facility utilization (Fink et al. 2015, Gabrysch et al. 2019). A substantial fraction of these deliveries happens at primary care facilities (Campbell et al. 2016), which often lack basic capacity to manage complications arising during delivery. The poor outcomes at primary facilities are due to lack of experienced and specialized staff, limited supplies and poor access to surgical and emergency services that are typically available in hospitals (Orwa et al. 2023).

In recognition of these challenges, the ‘Lancet Global Health’ Commission on High Quality Health Systems in the SDG Era (the ‘Quality Commission’) proposed Service Delivery Redesign—a reorganization of health systems, to optimize outcomes by ensuring that the right care is provided at the right level of the system and by the right provider (Kruk et al. 2018). Kakamega County implemented MNH SDR through a phased approach. The intervention aimed to shift all maternal deliveries from under-resourced primary care facilities to hospitals, enabling primary care facilities to focus on high-quality antenatal and postnatal care, and on linking women to hospitals for delivery care (Croke et al. 2022).

A 2019 feasibility assessment of the MNH landscape in Kakamega identified a number of gaps in a mother's journey through the health system, including poor availability and coordination of affordable emergency transport options, inadequate maternity bed capacity and critical hospital infrastructure, and shortages of skilled medical officers and obstetricians capable of delivering timely, advanced and dignified care to mothers (Nimako et al. 2021). These results were fed back to the Ministry of Health (MoH), the Kenya Council of Governors (CoG), and the Kakamega County leadership, including the Governor, Cabinet members, and senior officials of the County Department of Health.

**Figure 1:**
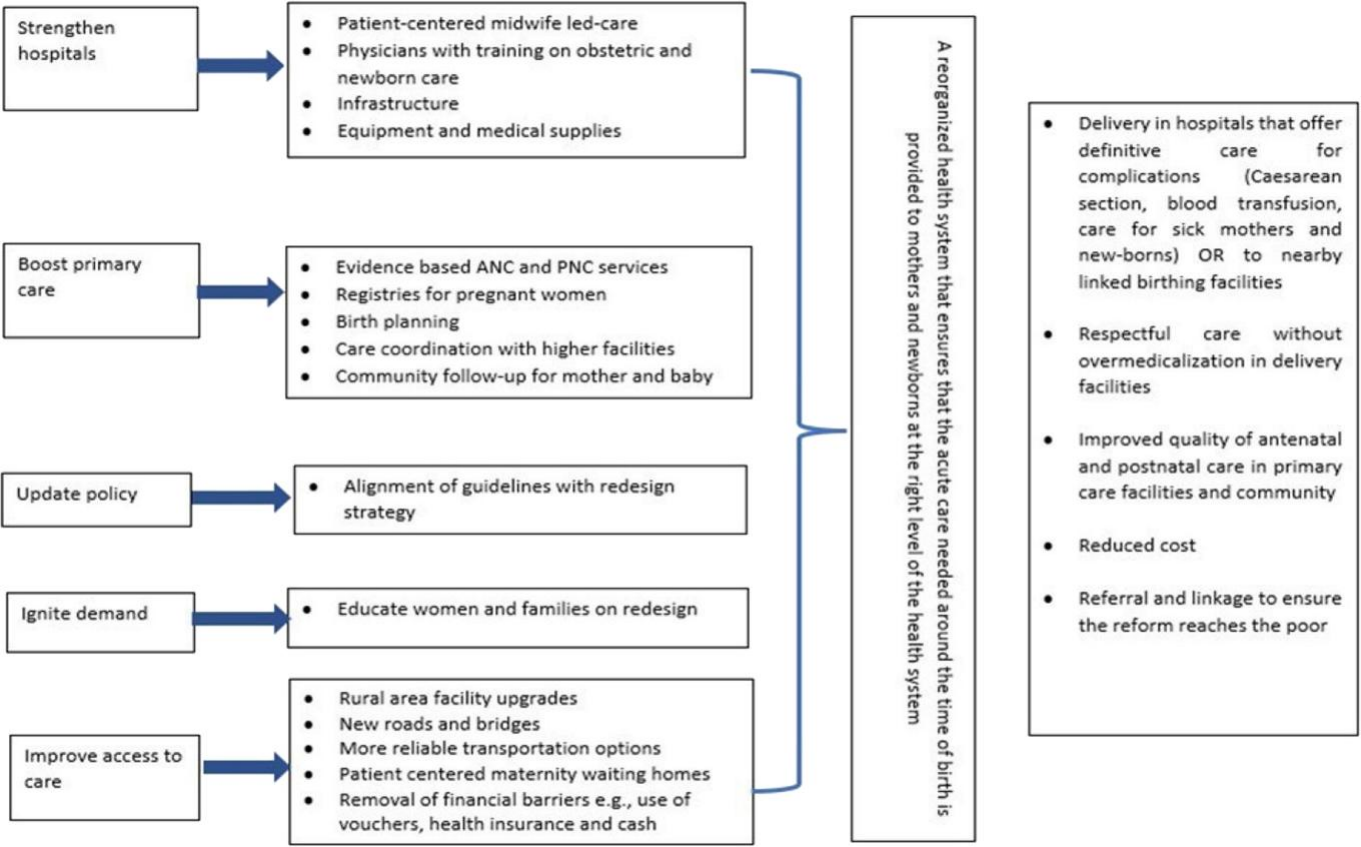
Overview of the SDR model in Kakamega County, Kenya. Illustrates the structural reorganization of maternal and newborn health services, showing the referral pathways between community units, delivery hubs, and county referral hospitals.

Consequently an SDR theory of change was collaboratively developed by the Kakamega County Department of Health, implementing partners, and donors. Key inputs included strengthening hospital with additional specialist staff and equipment, updating relevant policies (i.e. around referral and facility financing), igniting population demand for hospital delivery, and improving access to maternal and neonatal care at hospital level, particularly through improved emergency transport (Fig. 1) (1). The envisioned causal pathway in this theory of change emphasizes a collaborative pathway, requiring concerted efforts from multiple stakeholders. Moreover, it assumes that local actors are the key agents of change and that engaging communities is critical to the success of SDR.

Subsequent discussions with these stakeholders informed pre-implementation activities focused on improving the SDR design, identifying resource requirements, and engaging implementation partners. The county government committed to a 50–50 cost-sharing plan wherein the county government would provide supplies, commodities and health workforce support. NGO implementation partners, with external funding, would provide infrastructural upgrades, health worker training, health financing technical assistance, and referral transport services.

By the time of this study, SDR had progressed from design to early implementation, with infrastructure upgrades, training, and referral mechanisms underway in selected sub-counties. Preliminary evidence from county and partner monitoring reports suggested perceived improvements in the quality of facility-based care and declines in home deliveries. However, concerns were also raised about access for women living far from upgraded hubs, specialist workforce shortages at the hospitals and availability of long-term sustainable financing for the reform activities. These early observations provided a backdrop for examining SDR implementation in a devolved and resource-constrained setting.

## Materials and methods

### Approach

**Figure 2:**
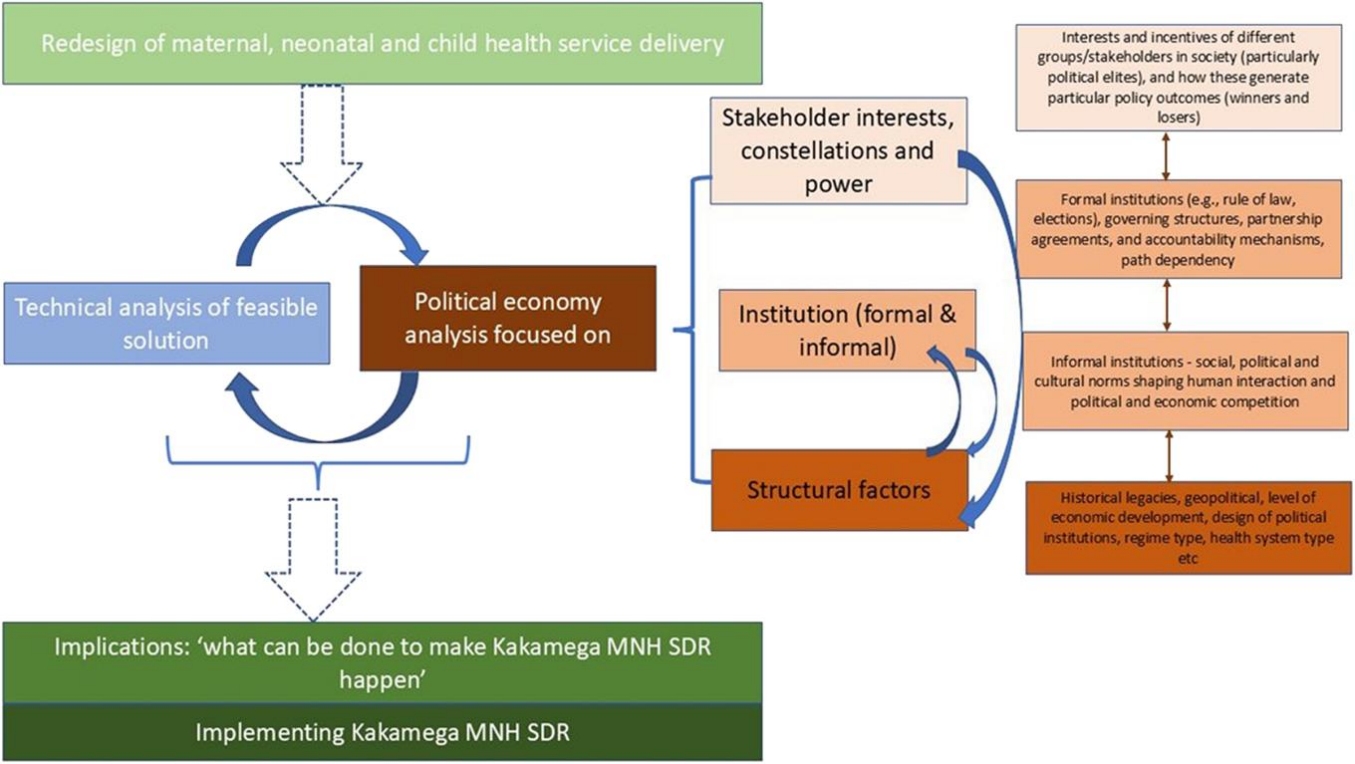
Analytical framework: stakeholder interests, institutions, and structural factors. Depicts the conceptual framework used for PEA, highlighting how stakeholder influence, formal/informal institutions, and structural context interact to shape reform outcomes.

Using a political economy lens, we sought to understand how health system actors, formal and informal power relations, and institutional structures influenced the SDR implementation process. Political economy is a diverse field with multiple methodological approaches. For our analysis, we developed a PEA analytical framework (Fig. 2) based on three existing theoretical approaches: the health policy triangle (Walt and Gilson 1994), Reich and Campos's framework (Reich and Campos Rivera 2024), and Wanyama and McCord's application of the political settlements approach to Kenya (Wanyama and McCord 2017). Walt and Gilson’s (1994) policy triangle reflects history, ideas and power through which actors exercise influence in the policy process, highlighting how the formal SDR agenda was shaped and executed within Kakamega County.

However, beyond the formal policy process, Campo and Reich's framework (Reich and Campos Rivera 2024) enables a deeper examination of the vested interests, power relations, and institutional structures that shaped the redesign trajectory. Yet, institutional structures are not only bound by formal by formal institutions but are also shaped by informal agreements and elite negotiations. Wanyama and McCord's application of the political settlements framework expands this analysis by allowing an examination of how resource allocation for SDR activities, bureaucratic behaviours of SDR actors, and accountability mechanisms to service providers and users determine how maternal and child health services are framed and implemented. By integrating these perspectives, this study highlights how structured policy processes and informal political dynamics collectively shape the implementation of SDR in Kakamega County (Nimako et al. 2021) (Fig. 2).

### Sampling

**Figure 3:**
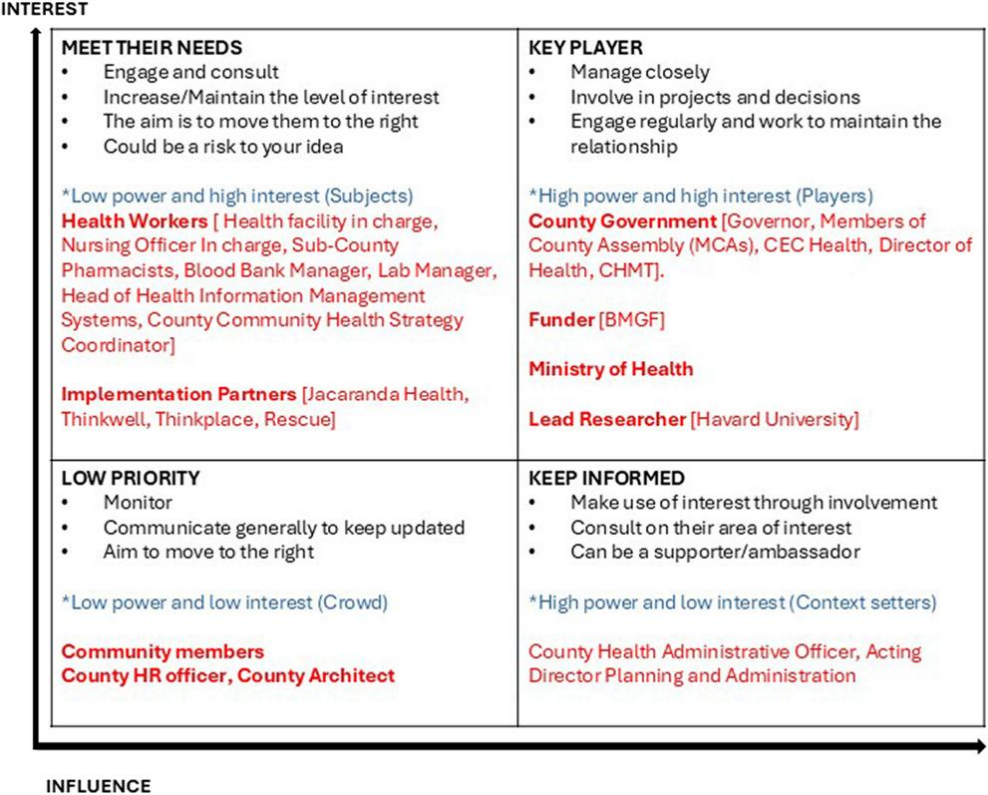
Stakeholder influence–engagement grid for SDR implementation. Shows the relative influence and engagement of key actors—community groups, health workers, political leaders, donors, and national institutions—across different phases of the SDR process.

We employed a phased approach in selecting the study population for this PEA. In Phase 1, we reviewed Kakamega County MNH and Consortium Partner reports to understand the MNH landscape (document review). Documents were obtained through targeted searches of county, ministry, and partner repositories, spanning 2018-2024. In Phase 2, we conducted a stakeholder analysis to map key SDR actors (stakeholder mapping; see Table 1 and Fig. 3). Phase 3 involved observing County Health Management Team (CHMT) and partner meetings (non-participant observation) to understand decision-making and implementation. In Phase 4, we interviewed 13 CHMT members, 5 development partners, and 5 implementation partners (key informant interviews). Phase 5 included focus group discussions (FGDs) with community health promoters (CHPs), women with recent facility or home deliveries, men, and traditional birth attendants. The interviews and FGDs lasted 45–90 min and followed semi-structured guides exploring SDR design, implementation, and service delivery experiences.

**Table 1:** Summary of main stakeholder and their role in SDR by stakeholder category.

Stakeholder group	Main actors	Expected role of actors in SDR
Kakamega County Government	Governor Members of County assembly CHMT	Lead reform prioritization, resource allocation, and implementation; leverage devolved authority to drive agenda-setting and adoption, ensuring political will and electoral support Increase staff numbers in hospitals, implementation of a digital blood tracker, facility construction, improve revenue collection through the NHIF, develop and implement the Facility Improvement Fund (FIF) bill, enhance antenatal care (ANC), and postnatal care (PNC) services
MoH CoG	Directorate of Standards, Quality Assurance and Regulation CoG Health committee	Provide national policy alignment and technical guidelines; support scale-up and coordination to ensure SDR aligns with UHC and SDG goals
Funder	BMGF	Fund feasibility studies, infrastructure upgrades, and evaluations; provide technical support to accelerate adoption and implementation while influencing sustainability strategies
Implementation partners	Jacaranda Health Think place Thinkwell Rescue	Ensure safe navigation and connection to care through training of health worker on MNH competences, facility construction and expansion of wards, provision of emergency referral transport systems, improve revenue collection through the NHIF, increase facility financial autonomy by developing and implementing the FIF bill, pilot human centred innovations, e.g. ticketing service
Researcher	Harvard University	Harvard researchers conducted feasibility assessments in Kakamega County and supported the development of the SDR strategy, identifying system gaps and testing assumptions to inform a model that shifts deliveries to better-equipped hospitals
Health workers		Deliver hub-based MNH services; participate in training and implementation, enhance ANC and PNC services
Community members	Mothers Other close community members	Demand quality, accessible care; provide feedback to shape implementation

Provides a description of the main SDR actors and their role in the SDR reform.

This multi-phased approach enabled a comprehensive understanding of the reform process by integrating diverse stakeholder perspectives (Table 2).

**Table 2:** Summary of study population and sampling.

Approach	Areas of inquiry	Sample frame
Document analysis	Framing of SDR, actor dynamics, and policy characteristics	SDR feasibility assessment reports Partners’ progress reports Partner meeting minutes
Stakeholder analysis	Stakeholders’ priorities, motivations, and influence	Kakmega County Government MoH CoG Donors Implementation partners Health workers Community members
Quarterly partner meetings	To bring the implementation partners together to create a shared vision of service delivery design aligned	SDR consortium partners 6 rounds of non-participant observations of delivery hubs from 2021 to 2023
Key informant interviews	Framing of SDR, the role and influence actors Frequency and nature of engagement of actors Level of cohesion with respect to SDR Public participation and community accountability	13 CHMT members 18 health workers 5 development partners 5 implementation partners
FGDs with the community	Framing of the SDR, how the community members were engaged, public participation, perceived benefits, and obstacles of the SDR to the community	5 FGDs with CHVs women with recent deliveries women with home deliveries men TBAs/birth companions

Provides an overview of the number and type of participants included in interviews and observations.

### Data collection

Our document analysis aimed to answer two questions: First, how was SDR framed and presented to key actors? Second, who were the key actors involved in the redesign, and what were their roles? We reviewed county documents and official communication between the county Department of Health and SDR implementation partners to understand the framing of SDR and identify key stakeholders. The documents reviewed included SDR-related Memoranda of Understanding (MOU) at the county level, implementing partner reports (e.g. Jacaranda Health monthly reports), County Integrated Development Plans, Annual Development Plans, Annual Work Plans, Program Based Budgets, and the Medium-Term Expenditure Frameworks.

Our stakeholder analysis followed a systematic approach (Künzler 2016) to identify actors through the four dimensions of interests, attitude, power, and/or influence. Based on this methodology, we conducted the following three main steps: (i) developing a checklist of all potential stakeholders; (ii) identifying each stakeholder's interest and attitude towards the SDR reform; and (iii) evaluating the degree of involvement of each stakeholder. Findings from this stakeholder analysis informed the development of interview and observation guides, which were subsequently used to explore key actors’ perspectives, influence, and engagement in SDR implementation.

The key informant interviews and FGDs focused on the framing of SDR, the role and influence of various actors on the design and implementation of SDR, the frequency and nature of engagement of actors, the perceived benefits of and obstacles to SDR, and levels of cohesion regarding public participation and community accountability. These areas of inquiry were tailored to the different stakeholder groups based on their expertise and role in the implementation process. The interview guide was iteratively refined to ensure emerging issues and insights were captured effectively.

### Data analysis

Our analysis was guided by the political economy framework depicted in Fig. 2, which examines stakeholder interests, formal and informal institutions, and structural factors shaping implementation. To capture power dynamics within communities, we analysed how gender roles, social hierarchies, and proximity to decision-making influenced experiences of SDR. This aligns with our political economy lens, which views power as relational and exercised both between institutions and within communities. The framework analysis drew upon: (i) ‘a priori’ issues (informed by the original research aims and incoporated in the interview guides), (ii) emergent issues which were raised by the respondents or otherwise emerged during data collection, and (iii) analytical themes arising from the recurrence of views or experiences that were interpreted as significant and relevant to SDR implementation.

We used an abductive approach consisting of iterative data interpretation cycles/analysis/reinterpretation/analysis. Document reviews and stakeholder mapping were analysed thematically to generate preliminary insights, which then informed coding of interview and focus group data. Interpretation and synthesis were conducted concurrently across sources, ensuring triangulation and complementarity across data sources.

## Results

We begin by outlining the MNH SDR implementation timeline and key features to provide context for the results. We then present findings under seven thematic areas adapted from the three main conceptual categories of our PEA framework.

First, we describe actors’ roles, power relations, and their position and influence on SDR implementation processes. Secondly, we describe how formal and informal institutions shaped actors’ interactions and political priorities. Lastly, we present relevant structural factors, which include the evolution and framing of SDR and how ideologies and values influenced its adoption. Finally we reflect on how SDR implementation has affected the availability of health resources and finance within the county.

### Stakeholders’ power and interest

#### Position of service delivery redesign actors

The stakeholder analysis examined actors involved in SDR design and implementation across global, national, county, facility, and community levels. Figure 3 presents the distribution of stakeholders by their relative influence and interest in SDR implementation, while Table 4 summarizes their evolving roles and power relations across different phases of the reform. In this analysis, *interest* refers to stakeholders’ level of engagement or commitment to SDR, rather than the degree to which they are affected by its outcomes, while *influence* indicates their ability to shape decisions or outcomes.

As shown in Fig. 3, the high-interest/high-power quadrant included funders and global technical partners [Bill and Melinda Gates Foundation (BMGF) and Harvard University], national actors (the MoH and CoG), and the County Government of Kakamega. These actors strongly supported SDR and were instrumental in shaping its design, mobilizing resources, and aligning policy priorities. The Governor and the County Executive Committee Members for Health were especially influential in framing SDR as a political and developmental priority, though their engagement was primarily strategic and high level.

Actors with moderate influence included implementation partners—particularly Jacaranda Health, which led local technical coordination and community sensitization—and county health managers who oversaw daily implementation. Health workers also occupied a middle ground: while their representative bodies had limited roles in reform design, frontline providers were essential to SDR's operational success.

The low-influence/high-interest quadrant comprised community members, traditional birth attendants, and CHPs. Although deeply affected by the reform, these groups had minimal capacity to shape decisions, reflecting persistent asymmetries in power and information. Their buy-in was critical but constrained by limited understanding of SDR's objectives and by structural barriers such as transport, distance and service costs.

Overall, the analysis revealed that no single stakeholder dominated decision-making (Table 3). Most implementation decisions were made through consultative platforms involving government, funders, and implementing partners.

I would not say that one partner has overwhelming or all the powers to decide for one partner or the rest of the partners. So, in the course of implementation, we have evolved to the point where we have specific groups that were set to achieve our set objectives. We even have even peer meetings where the consortium partners come together and have a review of the activities and what has been achieved and the next steps and all that.

However, this consultative structure did not imply equal power. High-influence stakeholders including the Governor and County Executive for Health used budgetary control and political authority to fast-track SDR feasibility studies, approve partner engagements, and link SDR to the Governor's electoral legacy. Funders such as BMGF influenced the agenda primarily through financing and by positioning SDR as a model aligned with global quality-of-care goals. National institutions like the MoH and CoG reinforced legitimacy by ensuring policy coherence, but largely followed rather than initiated the reform direction.

So, I can say that it is likely that the CoG drove this. We were not fond of the idea of SDR at the MoH. And with this compelling case, and the CoG involvement, it really worked out.

Moderately influential actors including health workers and the implementing partners shaped the operational reality of SDR rather than overall design. Partners such as Jacaranda Health translated political ambitions into technical plans, training staff, and coordinating advocacy activities, while frontline health workers were interested in the practical feasibility of the new service delivery protocols.

After the launch a Service Delivery Redesign plan which required a feasibility study and a way on how to implement the study…to get data that can help us move from there. We went to Kakamega and we [MOH, Harvard Team] started doing sensitization: telling them [Implementing partners, frontline health workers] what we want to do and the kind of data we want to collect.

Community members, traditional birth attendants, and CHPs were interested in how SDR could improve health outcomes but had limited influence over its design or implementation. Their engagement was often limited to awareness sessions rather than active participation in planning or feedback mechanisms, highlighting the need for stronger mechanisms to translate community concern into meaningful participation.

The CHPs expressed a unanimous agreement that they have not been adequately included in discussions regarding the reform. The last meaningful engagement they had was back in February 2023, and it only involved nine community units. They highlighted the challenge of insufficient information flow as a major hurdle.

This uneven distribution of power shaped how priorities were set and whose concerns were most reflected in SDR design and rollout (Table 4).

**Table 3:** Overview of key SDR key events and implementation features.

SDR implementation components	Activities/description	Implementation outcomes
1. Create a conducive policy environment	Endorsement of SDR by two different County Governors over the implementation span of SDR Creation of technical working groups, engagement with CHMT around budget and work plans to support SDR	Successful management of governor transitions have enabled Phase 1 through Phase 2 SDR implementation from six technical working groups (Human Resources for Health, Health products and Technology, Health Financing, Quality of Care, Referral and Innovations) have been set up comprised of some of the SDR implementation partners and the Kakamega CHMT
2. Prepare facilities/infrastructural reorganization	Structural facility upgrades (expanded maternity wards, newborn unit) Construction of health facilities and strengthening of diagnostic capacity, equipment supplies and ensuring availability of medicines	Completed in two Level 4 hospitals Incomplete construction of one sub-county hospital (Butere)
3. Improve transportation	Design and deployment of urgent/emergency transportation models by Rescue.co Continuous monitoring of location and distribution of public and private emergency vehicles	Enabled in three sub-counties (Malava, Butere, and Lumakanda Sub-County) At the end of 2024, the rescue team handed the emergency service responsibility back to the county government
4. Relocate all deliveries to hubs	Shift all deliveries from primary care facilities to Level 4 ‘Maternity Centre of Excellence’ Delivery Hub Hospitals	Two designated Level 4 ‘Maternity Centre of Excellence’, i.e. Lumakanda Sub-County Hospital and Malava Sub-County Hospital Delivery shift memos delivered to health facilities in Malava and Lumakanda sub-counties
5. Engage communities	Creation of ticketing service for all MNCH services at Malava sub-county Use of CHPs as ambassadors for SDR	Ticketing service did not continue throughout the implementation period Limited engagement and communication with the CHPs regarding SDR activities

Outlines SDR implementation components, accompanying activities, and implementation outcomes.

**Table 4:** Stakeholder roles and power relations during SDR implementation.

Phase	Stakeholder	Interest	Influence	Actions
Agenda setting	Harvard University	Defining and co-design of the SDR reform	High	Defining the concept through the launch of the Lancet Commission
	Kakamega county government	Reduce mortality; gain electoral support	High	Allocated resources, endorsed feasibility studies, prioritized SDR
	MoH and CoG	Achieve national UHC and SDG targets; standardize MNH care	High	Provided policy guidance, aligned SDR with Linda Mama scheme
	Funders	Advance global health (SDG 3); justify investments	High	Funded feasibility studies, provided technical support
	Implementation partners	Improve MNH, secure funding	Moderate	Promoted and implemented promoted ‘Tutunze’ strategy
	Health workers	Provide quality care, professional development	Moderate	Supported if resourced
	Community	Access quality, respectful care	Low	Limited role by low autonomy
Adoption	Harvard University	Demonstrating pathways to maximize quality care and optimize health outcomes	High	Advocacy, political engagement to mobilize support for the reform, resource mobilization
	Kakamega county government	Showcase SDR leadership, electoral capital	High	Committed budgets, selected pilot hubs (e.g. Malava, Lumakanda)
	MoH and CoG	Ensure national policy coherence	High	Endorsed SDR
	Funders	Shape priorities via funding	High	Funded pilot hubs
	Implementation partners	SDR advocacy, sustained funding	Moderate	Sensitized communities, supported training
	Health workers	Avoid service disruption	Moderate	Supported adoption if trained and resourced, follow county directives
	Community	Access improved services	Low	Supported if access was assured, resisted if barriers persist
Implementation	Kakamega County government	Deliver structural improvements, electoral gains, donor support	High	Upgraded hubs (e.g. beds, blood trackers), phased rollout
	MoH and CoG	National oversight, scale-up potential	High	Ensure county-national alignment
	Funders	Demonstrate impact	High	Funded infrastructure, supported EmONC training
	Implementation partners	Oversee implementation activities	High	Trained providers, manage a consortia of implementation partners
	Health workers	Deliver quality care	Moderate	Implemented redesigned care
	Community	Receive quality care, respectful care, reduced mortality	Moderate	Used hubs if accessible, resisted if transport/trust issues persist
Sustainability	Kakamega county government	Maintain SDR gains, secure funding	High	Strengthen county systems, expand workforce
	MoH and CoG	Scale SDR nationally, sustain UHC gains	High	Support scale-up, provide funding/technical aid
	Funders	Ensure scalable, cost-effective model	High	Fund evaluations, reduce dependency
	Implementation partners	Advocacy role, sustained funding	High	Continue engagement, support local governance
	Health workers	Operate in strengthened system	Moderate	Support hubs if resourced, resist if underfunded
	Community members	Long-term quality MNH services	Moderate	Sustain demand if trust/access improve; revert to home births if not

Maps stakeholders’ interests, influence, and key actions across agenda setting, adoption, implementation, and sustainability phases.

In summary, SDR's agenda setting and adoption were primarily driven by county government and funders, with national institutions providing policy legitimacy. Decision-making authority was concentrated within the County Department of Health, where access to budgets, technical expertise, and donor partnerships shaped priorities. Funders and partners influenced the process by guiding the evidence framing and funding opportunities, while county leaders translated these opportunities into politically visible achievements. Health workers’ support increased after receiving training and resources, but community engagement remained ambivalent due to limited understanding of the reform and weak feedback channels. Ultimately, SDR implementation depended on how county leadership balanced fiscal constraints, their political interests, donor expectations, and community trust in decision-making processes.

### Formal and informal institutions

Formal mechanisms including a signed memorandum of understanding (MOU), technical working groups and county budget approvals established roles, responsibilities, and legitimacy for SDR implementation. However, these structures often proved slow to translate intent into action, so actors relied on relational coordination to maintain momentum: as one respondent noted:

The launch of the Lancet Commission was a high-profile event that included the then-Kenya's First Lady, our Secretary of State for Health, the Cabinet Secretary for Health; we had also the Director General for Health. And then, the top leadership for health in all the forty-seven countries.

Informal networks among county leaders, funders, and implementing partners often facilitated quicker decision-making, particularly when bureaucratic procedures were slow. After the presentation of the SDR feasibility assessement report, the Kakamega Governor (who was at that time Chair of the CoG) had become interested, gave the ‘green light’ to SDR and directed that funding be incoporated into the subsequent budget cycle.

The good thing is that the chairman of the Council of Governors comes from Kakamega, and the Secretariat felt ‘why don't we invest in the Governor to see if he could accept.’ And he really accepted, so we got, immediately, good will from a county to experiment.

Therefore, while formal instruments set the rules of engagement, it is the informal power in the form of political proximity and donor relationships that determined which projects moved first and who benefited. The donor support provided technical and financial resources which helped secure political buy-in but constrained local adaptation. As one respndent noted: ‘when the feasibility study area was chosen, there was no turning back. It may not necessarily have been ideal: it's a small area, but we felt if somehow, we can get data that can help us move from there, it will be fine’.

Furthermore, county political strategies reinforced patronage, as county resources were sometimes channelled into to visible projects. One example was the decision to construct a new Level 4 hospital in one of the Phase 1 sub-counties (Butere) rather than upgrade the existing Level 4.

Butere hospital is just within the home turf of the former governor, Governor A, and you see this was his project. He wanted at least to leave something back at home that he could be remembered with. So, his intention was to do that huge facility so that is stands out, big, and knowing that he wanted to make also a name… So that is also one thing that would have affected whatever good plans that this project would have gained.

A second example was high visibility ribbon-cutting events that consequently marked the launch of SDR in Kakamega County. The governor's political strategy was consistent with SDR adoption, as it could be presented in a way that aligned with his electoral goals:

And politics may change towards the end. You may have a different governor, but they may see that this may help the community and may end up supporting the program. But if health is not their priority, all this might change. If the candidate wants to get re-elected in five years, SDR might be something they can use to say, ‘I have achieved a lot’.

The actual SDR implementation critically depended on the framing of the reform. SDR was branded as ‘*Take care of us Kakamega*’ for local ownership, but this did not always translate to a proper understanding of its aims:

The respondents had a limited understanding of Tutunze Kakamega, with only vague knowledge about the initiative.

In the end, the balance between formal legitimacy and informal influence contributed to initial implementation progress but remained uneven over time. Although the MOU provided a blueprint detailing a phased SDR implementation, changes in design and execution were made in the actual implementation. For example, by the time the pilot ended, the county–partner cost-sharing (a ‘50%–50% share in investment’) left recurrent financing and operational responsibility unclear and the emergency transport system was handed back to the county. Without clear mechanisms for maintaining additional staff, essential equipment, and community engagement, momentum for SDR implementation weakened.

In summary, political strategies were central to how SDR was implemented. The Governor's influence shaped which facilities were prioritized and how resources were distributed, while informal alliances between county officials, funders, and implementing partners determined the pace and visibility of progress. These dynamics illustrate that political leadership and relational networks were as influential as formal institutional rules in driving implementation.

### Structural context

Historically, global health agendas and donor funding (e.g. from (World Health Organization, United Stated Agency for International Development) have shaped Kenya's health policies over the years, fostering reliance on external resources (Ochieng'Opalo 2022). This influenced SDR's adoption as national and county elites, notably the CoG and the MoH, aligned the reform with global quality-of-care and SDG3 priorities to attract technical and financial support. As one official explained,

The implementation requires a lot of funding, specially, on health personnel because we needed more staff and we needed a lot of reorganization within the county to ensure that when mothers come, they can get the advanced care they need immediately: they don't have to wait, they have ways of getting blood or a caesarean section.

In Kenya, health is the largest devolved service which further complicated SDR's role in the structural landscape. While the devolution system expects county governments to adapt national health goals locally, it also creates political incentives for governors and MCAs to pursue visible, high-profile projects for their own gain. New health projects and initiatives, such as SDR, are therefore politically attractive as they bring tangible achievements, e.g. facility constructions and upgrades that in particular are visible issues for voters but enhance political capital. County health leaders must navigate these tensions to assert authority over SDR implementation while managing national oversight.

During Governor's A tenure, Matungu facility was not that much, we couldn't see a lot of activities in terms of development, towards that facility. But then Governor B, came in, and his home turf is in Matungu. So, things are beginning to shift now, and you will a lot of developments there now being taken to facilities around that area.

Devolution thus allowed SDR to proceed but also introduced uneven implementation shaped by electoral considerations.

From a national health policy perspective, while SDR's emphasis on upgrading maternity hubs was framed as consistent with SDG3, it reportedly conflicted with the primary health care (PHC) and Universal Health Coverage (UHC) principle of delivering care closer to people. One MoH official observed,

You are negating the policy of the Ministry of Health of access, of what you call Universal Health Coverage: let's make sure there is access for all mothers for every care in dispensaries, in low-level facilities. So, I had to talk to my supervisors, then, [to tell them] that we are doing this case [SDR], and that it is an experiment. And they agreed reluctantly.

Kakamega's SDR was generally perceived as a pioneering reform driven by political will, donor support, and evidence-based policymaking, but whose sustainability required aligning global evidence with national policy priorities and managing political dynamics.

SDR was first owned by the politicians before coming to the technical people, who took a lot of time to learn what SDR is all about. They were fighting it in the first instance, in the name of: okay, UHC says services should be closer to people, but SDR is taking deliveries farther away from the people. So, if ownership can begin at the director level, at the CHMT level and it goes upwards, I think such a project has a high chance of success, as opposed to one that starts from a politician.

The decision to align SDR more closely with global quality agendas rather than PHC was brokered through negotiation between the CoG, MoH, and external partners, prioritizing technical excellence over access equity. This trade-off enhanced donor and political buy-in but was seen as potentially reducing health access for women in remote areas.

Despite SDR's alignment with local political strategy, the actual implementation was plagued with systemic challenges. SDR's lack of a dedicated budget, coupled with delayed national disbursements and chronic workforce shortages including a 2024 doctors’ strike (https://theconversation.com/kenyan-doctors-strike-the-government-keeps-failing-to-hold-up-its-end-of-the-bargain-228294) which exacerbated existing supply constraints. As a result, concerns about the sustainability of SDR were evident from the onset but remained unresolved throughout implementation.

I think SDR was a little bit ambitious. I mean, we looked at the global standards, but then the maturity of a local government like Kakamega to meet those global standards, I think it wasn't really, we were not yet there, the county was not yet there. So, the county made some commitments that the county struggled to deliver on.

Overall, the political economy of SDR reveals how stakeholder interests, institutional norms, and structural constraints interacted to shape the implementation process. Political leadership and donor alignment enabled early achievements, but weak community engagement and fiscal dependence limited sustainability. Using a PEA lens helps explain these dynamics by showing how formal authority, informal relationships, and political incentives intersected to both enable and constrain reform. Understanding these interactions points to practical entry points for future reforms: strengthening county-level financial autonomy, embedding community participation mechanisms, and aligning political incentives with service equity.

## Discussion

This paper examines the structural, contextual, and institutional factors shaping a MNH service redesign in a rural Kenyan County. Our findings extend existing political economy scholarship by demonstrating how local autonomy shapes health reform processes, the critical role of informal relationships in navigating formal bureaucratic barriers, the politics that prioritize reform visibility over sustainability, and the balance between donor dependency and local ownership.

A key insight emerging from this research is the central role of informal rules and relationships in sustaining implementation momentum when formal structures proved inadequate. Kakamega's devolved authority enabled commitment of resources to SDR pilot sites amid chronic systemic constraints of workforce shortages and infrastructure gaps. County leaders then leveraged donor funding and partner expertise, framing SDR as part of the Governor's health legacy for the county. SDR was consequently embedded in county health budgets, led by the Health and Sanitation Committee and the County Assembly, while the donor and implementation partners eased the associated financial and technical burdens.

While formal exercises, such as the 2019 Kakamega SDR feasibility assessment (Nimako et al. 2021), provided conceptual structure and guidance, informal mechanisms allowed quick problem-solving and flexibility. This pattern reflects broader evidence that relationships, shared sense-making, and collaborations enhance responsiveness and adaptability in service delivery, particularly where bureaucratic structures are slow or ineffective. We extend this broader literature by demonstrating how informal rules should be understood as part of everyday governance rather than gaps in formal systems, and why they are essential for understanding why reforms thrive or stall.

Our research reveals tensions in how political incentives shaped SDR implementation. County leaders prioritized highly visible, voter-friendly interventions characterized by ribbon-cutting ceremonies over essential health systems investments like routine infrastructure maintenance, adequate staffing levels, and community engagement systems. This patten was reflected in resource allocation: newly constructed surgical theatres and newborn units that remained non-functional due to insufficient recurrent budgets for supplies and specialized staff. These findings illuminates why SDR endorsement flourished while its rollout faltered-because the reform served political ambitions through optics and credit-claiming opportunities (Ochieng'Opalo 2022), without securing the ongoing commitments required for lasting change. Such dynamics reflect well-documented inefficiencies when political considerations affect project implementation (Tsofa et al. 2023), where elected officials use new health initiatives to garner voter support, donor goodwill, and leverage with national authorities (Kamau et al. 2024).

The findings also reveal a misalignment between global ambition and local capacity. SDR was designed to align global quality-of-care and SDG3 priorities, yet county-level fiscal space and workforce capacity were limited. The county's ability to commit resources to pilot sub-counties demonstrated devolution's potential, yet chronic workforce shortages, infrastructure gaps, and delayed national funding transfers repeatedly disrupted implementation, a recurring challenge in Kenya's devolved health system (Masaba et al. 2020). The policy environment further complicated matters, as overlapping national-county mandates, including newly passed Kenya's UHC and PHC legislation in 2023 (Chuma and Okungu 2011, Mwai et al. 2023), required ongoing negotiation with national authorities. Some stakeholders perceived SDR as diverging from national PHC priorities, yet historical centralization patterns meant that the MoH provided technical guidelines with limited implementation oversight, creating accountability gaps that left counties navigating reforms with insufficient support. A political analysis helps identify how subnational governments navigate global agendas within local resource and political constraints and provides lessons on how to design more realistic, context-sensitive interventions.

Power asymmetries also shaped implementation processes and early outcomes. County governors and senior officials held most decision-making authority, while national actors provided legitimacy but exercised limited oversight. Community members and health workers, although highly invested in the success of SDR, had little influence over its design. CHPs were particularly critical: they play a vital role in linking health services to community needs but their limited engagement posed a barrier to reform success. Evidence from other contexts shows that improving reproductive, maternal, newborn, and child health requires effective community engagement (Lassi et al. 2016). Despite low community trust rooted in access uncertainties, political support in the county government supported rapid decision-making, driven by political incentives. At the same time, SDR's external dependency posed risks, consistent with broader health system devolution challenges in Kenya (Tsofa et al. 2023).

Finally, while external resources can catalyse innovation, they also reduce pressure for local resource mobilization and long-term planning. Without a dedicated SDR budget line and with operational needs exceeding total health allocations, transitioning from donor to county funding appears unlikely in the country's current fiscal environment. This shortfall underscores structural challenges, including maintenance of surgical theatres, functional neonatal units, and blood transfusion services, which require consistent budget allocations. While donor funding and partner expertise enabled SDR's launch by easing financial and technical burdens that would have been challenging for the county alone, our research documents how this external dependency created sustainability risks and dependency in devolved systems.

Applying a PEA lens to these findings offers practical guidance for future reforms. First, political mapping and stakeholder analysis should be continuous processes, recognizing that alliances and influence shift over time. Second, establishing joint coordination platforms—bringing together county officials, partners, and community representatives—can bridge formal and informal governance, combining accountability with adaptability (DiMartino et al. 2018). Third, building county fiscal resilience and phased transition plans can reduce dependency on donor funds and short-term political priorities. Finally, embedding community engagement and transparency mechanisms within reform governance can strengthen trust, legitimacy, and sustainability.

Based on our findings, we offer specific guidance for teams undertaking major health system reforms. First, mapping and leveraging informal networks should begin early in the reform process. Stakeholder mapping must explicitly identify informal relationships and communication channels alongside formal structures, engaging respected informal leaders such as, community coordinators, and peer networks as implementation partners from the design phase. Rather than attempting to replace informal practices with bureaucratic processes, reform teams should create mechanisms that formalize successful informal practices that are already functioning within the system.

Second, addressing the visibility-sustainability gap resuires strategic political engagement: routine operational costs such as staffing and infrastructure maintenance can be frames as part of the Governor's health legacy, ensuring that essential but less visible inputs are supported alongside high profile outputs like facility launches.

Third, reforms must be designed for fiscal realism from the outset. This requires calculating full lifecycle costs including recurrent expenses before launch and identifying sustainable domestic financing mechanisms during pilot phases rather than after donor exit.

Fourth, navigating national-county dynamics proactively is essential in devolved systems. Establishing formal coordination mechanisms with national authorities early helps clarify how county innovations align with national priorities and reduce perceptions of divergence. Reform processes should be carefully documented to facilitate national adoption, and county leaders should advocate for clearer delineation of national oversight versus county autonomy in implementation.

Finally, community engagement must be treated as critical infrastructure requiring sustained investment. This means allocating dedicated budgets for CHP involvement in planning and ongoing implementation, recognizing that community trust building requires consistent investment rather than one-off sensitization activities. These recommendations recognize that PEA is not merely an analytical tool but should inform practical reform design—acknowledging political realities while creating structures that channel political incentives towards sustainable health outcomes.

## Conclusion

The multifaceted complexities surrounding the implementation of SDR reveal how structural, contextual, and institutional factors significantly shape health system reforms. Devolution and political will enabled implementation flexibility, but donor dependence, limited resources and weak community engagement exposed the reform's fragility. Informal relationships among actors helped sustain momentum where formal systems lagged, yet reliance on personal networks made implementation vulnerable to leadership and funding changes.

SDR's implementation requires more than technical design; it requires aligning global standards with local capacity, strengthening county financing, and embedding community participation in decision-making. Applying a political economy lens helps move from describing barriers to designing context-sensitive strategies and offers practical entry points for more adaptive and equitable implementation. By addressing donor dependency, managing political risks, and centering community engagement, Kakamega can sustain MNH improvements and inform future health system reforms across other devolved and resource-contrained contexts.

## Data Availability

Data will be made available after the SDR impact evaluation study is complete

## Reflexivity statement

The multidisciplinary authorship team spans health systems, political economy, and implementation research, representing early-career to senior investigators across Kenya, the United Kingdom, and the United States. Six authors are Kenya-based, bringing deep experience in qualitative and policy research and engagement with communities and policymakers in sub-Saharan Africa. Collaborators from UK and US institutions contribute comparative perspectives on health systems reform. This diversity in expertise, geography, and seniority strengthened reflexive analysis of power, voice, and context. We acknowledge the vital contributions of local researchers, community participants, and implementers whose insights shaped this study.
